# Letter to the editor: SARS-CoV-2 detection by real-time RT-PCR

**DOI:** 10.2807/1560-7917.ES.2020.25.21.2000880

**Published:** 2020-05-28

**Authors:** Trestan Pillonel, Valentin Scherz, Katia Jaton, Gilbert Greub, Claire Bertelli

**Affiliations:** 1 Institute of Microbiology, Lausanne University Hospital and University of Lausanne, Lausanne, Switzerland

**Keywords:** SARS-CoV-2, COVID-19, Molecular diagnostic, real-time RT-PCR

**To the editor:** The rapid development of open diagnostic methods for the detection of severe acute respiratory syndrome coronavirus 2 (SARS-CoV-2) has been key to build capacity for efficient molecular diagnostic tests in laboratories worldwide. These methods based on real-time RT-PCR were recognised as reference protocols since mid-January, at the onset of the pandemic in China, and relayed – although not validated – by the World Health Organization [1]. We would first like to acknowledge the contribution of Corman et al. [2] who were among the first to provide primers and probes for three genes (E, N and RdRp), that have been widely implemented across the globe to tackle the coronavirus disease (COVID-19) pandemic. They recommended the use of the PCR targeting the E gene followed by confirmation with RdRp primers combined to a SARS-CoV-2 specific probe.

Our diagnostic laboratory also implemented and used the above-mentioned assays [2] throughout the first months of the pandemic on its automated platform [3]. RNA was extracted from clinical samples with the MagNA Pure 96 System (Roche, Basel, Switzerland) and the real-time reverse-transcription PCR (RT-PCR) was performed on a QuantStudio 7 system (Applied Biosystems, Waltham, United States). RT-PCRs targeting E and RdRp were used routinely in parallel for 893 samples. In 115 cases with positive amplification by both RT-PCRs, the RdRp assay showed a significantly (paired Wilcoxon rank test, p value < 0.001) higher average cycle threshold (CT) (25.0 CT, interquartile range (IQR): 24.6–27.9) than the E target (22.6 CT, IQR: 19.3–25.8). Furthermore, positive E and negative RdRp results were obtained in 10 cases (1%), triggering further investigations, since patients were unlikely to be infected by other SARS-related viruses that can also be amplified in the E assay. Conversely, no negative E and positive RdRp RT-PCR results were observed.

After careful review of the initial manuscript and analysis of SARS-CoV-2 and other coronavirus sequences, it appeared that the proposed RdRp reverse primer contained an incorrect degenerate base (S), that does not match with the SARS-CoV-2 RNA sequence, as shown in the alignment of Corman et al. Figure 2 [2]. Indeed, the proposed RdRp_SARSr-R sequence (CARATGTTAAA**S**ACACTATTAGCATA, R = [AG], S = [GC]) does not match any of the 1,623 SARS-CoV-2 complete genome sequences publicly available in the National Center for Biotechnology Information (NCBI) database as at 7 May 2020. Instead, the corrected RdRp_SARSr-R2 sequence (CARATGTTAAA**R**ACACTATTAGCATA, R = [AG]) should allow to amplify SARS-CoV-2 genetic material, including loosely related bat and human sequences, with improved efficiency. Two additional nucleotides in the pan-Sarbecovirus probe RdRP_SARSr-P1 (CCAGGTGGWAC**R**TCATCMGG**T**GATGC, W = [AT], R = [AG], M = [AC]) should also be replaced (RdRP_SARSr-P1b, CCAGGTGGWAC**M**TCATCMGG**W**GATGC, M = [AC], W = [AT]) to improve similarity with the SARS-CoV-2 and bat coronavirus genetic sequence while retaining the pan-Sarbecovirus compatibility. These observations based on in silico alignments should be confirmed by wet-laboratory experiments, but they could explain the lower sensitivity of the RdRp RT-PCR also shown by Vogels et al. [4] and point towards potential improvements.

As the pandemic spreads, many laboratories worldwide, including in low-resource countries that may not rely on expensive commercial kits, implement routine diagnostic tests. Thus, we think that such information is critical to ensure a proper detection of SARS-CoV-2 infections, allowing efficient isolation and preventing further transmission of the virus.
